# Effects of 1, 2, 4-Triazole Additive on PEM Fuel Cell Conditioning

**DOI:** 10.3390/membranes10110301

**Published:** 2020-10-22

**Authors:** Nana Zhao, Zhiqing Shi, Régis Chenitz, François Girard, Asmae Mokrini

**Affiliations:** 1 Energy, Mining & Environment Research Centre, National Research Council Canada, 4250 Wesbrook Mall, Vancouver, BC V6T 1W5, Canada; francois.girard@nrc-cnrc.gc.ca; 2 Automotive and Surface Transportation Research Centre, National Research Council Canada, 75 de Mortagne, Boucherville, Québec, QC J4B 6Y4, Canada; Regis.Chenitz@cnrc-nrc.gc.ca

**Keywords:** proton exchange membrane (PEM) fuel cell, 1, 2, 4-triazole, additives, contamination, conditioning, melt blowing processing

## Abstract

Melt processing is one of the essential technologies for the mass production of polymer electrolyte membranes (PEM) at low cost. Azoles have been widely used in PEM to improve their conductivity at a relatively low humidity and recently as bifunctional additives in a melt blowing processing for PEM mass production. In this work, we attempted to assess the effect of 1, 2, 4-triazole additive in membranes and in catalyst layers on PEM fuel cell conditioning. Various characterization tools including electrochemical impedance spectroscopy (EIS), cyclic voltammetry (CV) and conditioning with constant current were applied to diagnose the temporary electrochemical reaction effect and the permanent performance loss caused by the triazole additives. It was found that triazole additives in membranes could migrate into the catalyst layers and significantly affect the open circuit voltage (OCV) and the conditioning. The effect could be partially or completely removed/cleaned either through longer conditioning time or via CV cycling, which depends on the amount of additives remaining in the membrane. The findings provide valuable scientific insights on the relevance of post treatment steps during membrane production and overcoming fuel cell contamination issues due to residual additive in the membranes and understanding the quality control needed for fuel cell membranes by melt blowing processing.

## 1. Introduction

Proton exchange membrane fuel cell (PEMFC) technology has been widely considered as the next revolution in renewable energy due to its high theoretical energy efficiency and zero carbon emissions at the point of use [1]. However, two major limiting factors, cost and durability, limit large-scale implementation of fuel cell technology for use in transportation applications [2]. The proton exchange membrane (PEM) was identified as one of the most expensive stack components, which is about 8% of the total stack cost if annual production rates (APR) is 500,000 fuel cell vehicle (FCV)/year [3]. Melt processes represent one of the most interesting technologies for the mass production of homogeneous thin polymer films at low cost. The processes could not only provide a mechanical enhancement through chain orientations following extrusion-stretching, but also reduce the manufacturing cost [4]. The azole family compounds such as 1, 2, 4-triazole, imidazole and benzimidazole have been widely used as potential bifunctional additives in a melt blowing processing [5]. They act as sulfonic acid groups (-SO_3_H) protection and a melt processing aid to assist proton conduction by Grotthus mechanism under anhydrous/high temperature conditions [6,7,8]. However, excess additives in a membrane must be washed away in the final manufacturing step because azole family compounds could cause strong adsorption on the surface of the catalyst, which may lead to losses in electrochemical activity of the electrodes and decreased fuel cell performance [9]. Extensive research has been done on contamination caused by fuel-side impurities such as CO, H_2_S, and NH_3_; air-side impurities, including NO_x_, SO_x_, and CO_x_; and volatile organic compounds [10]. There has nevertheless been much less research on the impact of membrane additive contamination on fuel cell performance, especially organic membrane processing additives. The 1, 2, 4-triazole was selected as a model of imidazole family because it is commonly used in melt processing [9,10,11,12,13]. Its strong parallel adsorption on metal has been proven as a well-known steel corrosion inhibitor [14]. In this work, we attempted to understand the factor of 1, 2, 4-triazole additive migrating from membrane to catalyst layers and how the additive in catalyst layers affects fuel cell performance. 

This work is an investigation of the effects of the contamination of 1, 2, 4-triazole in membranes or cathode electrodes on fuel cell conditioning. Electrochemical impedance spectroscopy (EIS), cyclic voltammetry (CV), and conditioning with constant current were applied to diagnosis the temporary electrochemical reaction effect and the permanent performance loss by additives. X-ray photoelectron spectroscopy (XPS) and UV-Vis were carried out for post analysis to identify additive migration. All of these characterization results were combined to understand the effects of 1, 2, 4-triazole on PEMFC performance, which is an effort toward assessing the relevance of post-processing additive removal step, overcoming fuel cell contamination problems in the case of residual additive and understanding the quality control needs for melt blown membrane fabrication.

## 2. Materials and Methods 

### 2.1. Membrane−Electrode−Assemblies (MEAs) and Single Cell

*Membrane:* Membrane samples of Nafion^®^ NRE-211 (NRE-211, DuPont, Wilmington, DE, USA) were manufactured by DuPont and purchased from Fuel Cell Store, USA. NRE-211 is a single layer and its thickness is 25.4 μm, with ~1.0 mmol g^−1^ of ion exchange capacity (IEC). NRE-211 contains no additives. The membrane with 1, 2, 4-triazole was fabricated via a melt blowing process using Nafion^®^NR40 (1000EW) as ionomer and 1, 2, 4-triazole as additive without an acid washing step [13]. 10 wt% of additive in ionomers pellets-additive blends was incorporated before processing. The melt blown fabricated membrane’s thickness is ~25 μm, with ~1.0 mmol g^−1^ of IEC. 

*Gas diffusion electrode (GDE)*: Carbon paper based GDE with 0.3 mg cm^−2^ of platinum on carbon (40%) was purchased from Fuel Cell Store, USA. 

*Introducing Triazole into GDE:* GDE was treated by soaking a piece of 5 × 5 (cm × cm) standard GDE in 30 mL of either 1 ppm or 50 ppm of 1, 2, 4-triazole additive aqueous solution for 48 h before being dried by air blowing at room temperature (RT). The standard GDE without any pre-treatment was set as baseline GDE. 

*MEAs:* Membranes were inserted between two GDEs (either standard GDEs or triazole-introduced GDEs) and assembled directly into a fuel cell test fixture from Scribner Associates Inc. (straight flow channels and an active area of 25 cm^2^) without hot-pressing. The uniformity of the cell compression was tested using pressure-sensitive films (PRESSUREX^®^FILM, Ultra low film, 28-85psi, Sensor Products Inc., Madison, NJ, USA). The single cells were conditioned in a 100W fuel cell test station (Scribner 850C, Scribner Associates Inc., Southern Pines, NC, USA).

### 2.2. Fuel Cell Conditioning Protocol

The MEAs were preheated for 40 min at 68 °C under 100% humidity on both sides, with H_2_ (purity 99.999%) flow rate of 2 standard liters per minute (SLPM) at the anode, N_2_ (purity 99.999%) flow rate of 5 SLPM at the cathode, and without back pressure on both sides. After preheating, the MEAs were conditioned at either 10A or 20A, 100% RH, and 68 °C for 16 h, which the load current depends on fuel cell performance. H_2_ (purity 99.999%) flow at 2 SLPM and ambient air flow at 5 SLPM were used for the cathode and anode without back pressure, respectively, during conditioning.

### 2.3. In-Situ Electrochemical Impedance Spectroscopy (EIS)

To monitor the 1, 2, 4-triazole additive migration, in-situ EIS was conducted during conditioning (under direct current, 20A) by imposing a small amplitude alternating current (AC) signal to the fuel cell via the load. The frequency range is from 10 kHz to 0.1Hz. The voltage responses were recorded and decoupled by a built-in frequency response analyzer (FRA, Scribner 880).

### 2.4. Electrochemical Surface Area (ECSA)

ECSAs of the cathodes were measured by CV using a potentiostat (1287A, Solartron Analytical, Farnborough, UK). The anode and cathode were purged for 20 min with humidified H_2_ (0.5 SLPM) and N_2_ (0.5 SLPM), respectively. Voltammograms were then recorded using a 50 mV s^−1^ scan rate between 0.1 V and 1.2 V versus the anode under H_2_ (0.5 SLPM) and N_2_ (0.5 SLPM), respectively. The anode has been considered as a reference hydrogen electrode by deliberately neglecting its contamination to 1, 2, 4-triazole and resulting effect on electrochemistry of Pt nanoparticles toward H_2_/H_2_O.

### 2.5. Ex-Situ Characterization

UV-vis absorption spectra were recorded on a Varian 50 Conc UV-Visible Spectrophotometer (Agilent Technologies, Santa Clara, CA, USA). The film holder was used for membrane samples to record the spectra ranging from 200 nm to 800 nm.

X-ray photoelectron spectroscopy (XPS) was performed by an Omicron XPS System (Scienta Omicron, Taunusstein, Germany) using monochromatic (Al K alpha) X-ray source at 150 W in the pass energy (PE) mode (PE = 20 eV). All of the spectra were obtained under identical conditions. The pressure of the spectrometer was 5 × 10^−10^ and 5 × 10^−9^ mbar during the measurements.

## 3. Results and Discussion

### 3.1. Open Circuit Voltage (OCV) for Membranes with 1, 2, 4-Triazole Additive

**Figure 1:**
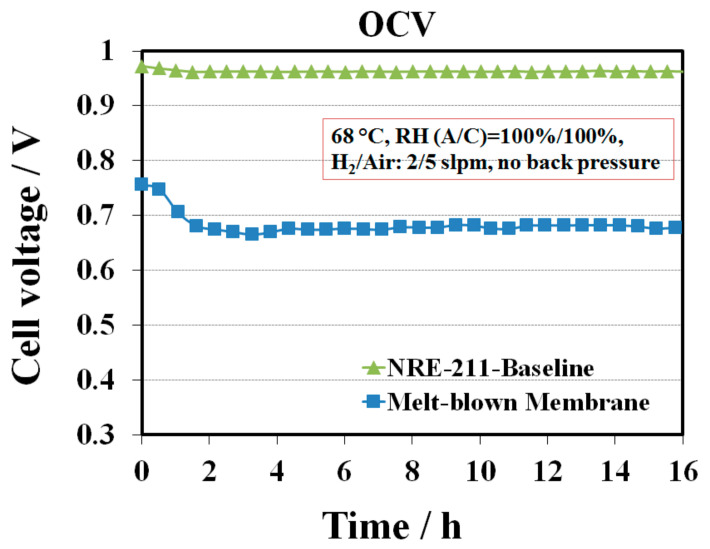
Open circuit voltage (OCV) profile of the melt-blown membrane as processed with 1, 2, 4-triazole additive and the baseline NRE-211 at 68 °C and 100% RH.

The melt blown membrane with 1, 2, 4-triazole additive was fabricated and assembled without any further post-treatment into a single cell to evaluate its fuel cell performance. Conditioning curve for this sample was not able to be collected since the cell performance was too low to draw any load. Alternatively, OCV hold testing was performed. Figure 1 shows a comparison of a single cell OCV profile between the melt blown membrane and the baseline NRE-211 at 68 °C and 100% RH. The OCV curve for the baseline sample was flat with only 1% variation in cell voltage compared to the initial voltage. In contrast, the OCV for the melt blown sample decreased during the first few hours, then slightly increased, and next kept constant over time. The lowest OCV points for the melt blown sample was at ~ 3.5 h with OCV of 665 mV. Compared to the OCV of the baseline in the same range of time (~970 mV), the OCV of this sample was ~300 mV lower, implying that the 1, 2, 4-triazole additive has a considerable impact on the cell performance. Generally, the OCV depends on the hydrogen permeation rate across the membrane as well as the mixed potential of the electrochemical reactions of Pt surface oxidation and the oxygen reduction reaction (ORR). Lower OCV value indicates either high reactant crossover and/or electronic short through the membrane, or poisoning of the catalyst or electrolyte [15]. In our case, the lower OCV phenomena could be explained by the contamination of the catalyst layers by the 1, 2, 4-triazole additive, as it likely migrated from the membrane into the catalyst layers, leading to a decrease in OCV in the first few hours. While the accumulated additive in the catalyst layers achieved its maximum value, the OCV started to slightly increase probably due to partial contaminant removal from the cell via convection. However, the real mechanism of additive migration is not clear. It is hypothesized that at the beginning of the MEA assembly, 1, 2, 4-triazole additive might migrate from the membrane into the catalyst layer and be retained at the Pt surface by forming covalent bonds between the nitrogen (N) in 1, 2, 4-triazole and the Pt atom causing the initial relatively low OCV [16]. MEA humidification during OCV test exacerbated the catalyst layers contamination afterwards because the additives could keep migrating along with the water from humidified gas streams. Meanwhile, a portion of retained 1, 2, 4-triazole could be washed out from the catalyst layer into the GDLs or released from the MEA by water. The amount of triazole remaining in the catalyst layer relies on the dynamic balance between the amount of additive migrated from the membrane and the amount of additive moved away from the catalyst layer. 

**Figure 2:**
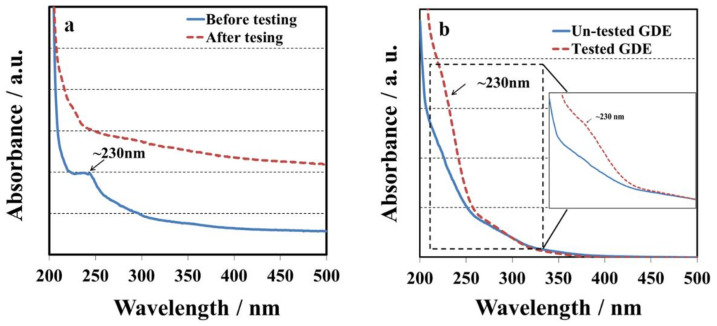
UV spectra of (**a**) melt blown membrane sample before and after OCV hold test; (**b**) UV spectra of the soaking solutions (ethanol/water mixed solvent) for un-tested and tested GDE.

The hypothesis can be supported by UV-vis spectra of the tested membranes and GDEs. 1, 2, 4-triazole is one type of heterocyclic compounds with the molecular formula C_2_H_3_N_3_, which has a five-membered ring containing two carbon and three nitrogen atoms and can easily be detected by UV spectra. Figure 2a shows the UV spectra of the membrane with 1, 2, 4-triazole before and after OCV hold test. There was one absorption peak at ~230 nm associated with 1, 2, 4-triazole before OCV hold test. However, the absorption peak at ~230 nm completely disappeared after 16 hours’ OCV hold test. The changes in UV spectra could be explained by the fact that 1, 2, 4-triazole moved away from the membrane during fuel cell testing. Although the absorption peak at ~230 nm disappeared after fuel cell testing, there might be some leftover species which were undetectable by the spectrometer. In order to further prove our hypothesis, the tested GDE on the cathode side and a standard GDE before testing were immersed in ethanol/water solvents (7: 3) for 12 h, and then their solution samples were examined by UV-vis. Figure 2b displays one weak absorption peaks at ~230 nm associated with 1, 2, 4-triazole diffusion to the soaking solution from the tested GDE, confirming that additive migration from the membrane into the catalyst layer occurs via convection of either humidified gas or water. For the soaking solutions with the un-tested GDE, the peak at ~230 nm could not be detected indicating the amount of triazole in catalyst layer is insignificant. However, trace amount of triazole could cause a significant impact on the OCV. 

**Figure 3:**
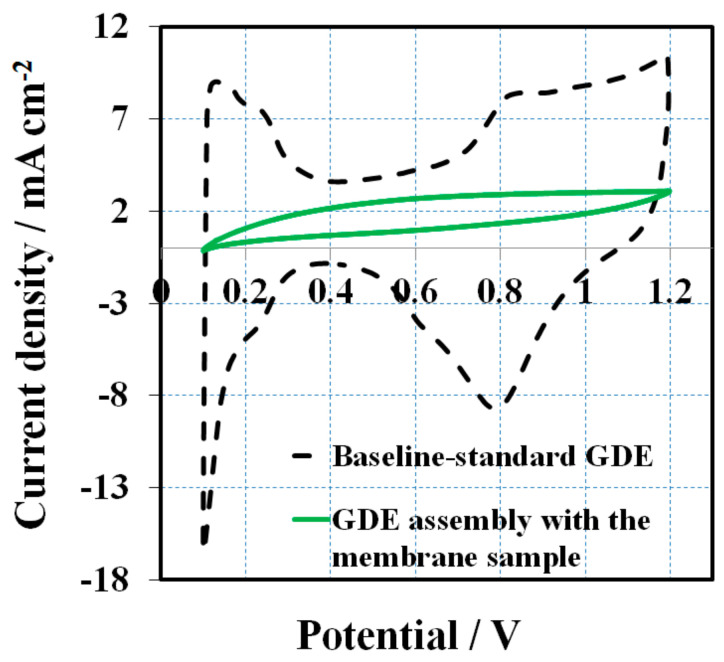
Cyclic voltammetry (CV) curves for the MEAs with the membrane sample and baseline at a scan rate of 50 mV s^−1^ and potentials of 0.1 to 1.2 V.

After a 16 h OCV hold test, CV scans were performed to compare the cathode side GDE against the baseline to determine if the additive had contaminated the cathodic catalyst layer. All of the GDEs before MEA assembly is assumed to have an identical CV (commercial standard GDEs from the same batch). Figure 3 presents the CVs obtained at a scan rate of 50 mV s^−1^ after the OCV hold test. The baseline CV (black dashed line) shows characteristic CV peaks for hydrogen adsorption/desorption of Pt electrode. The initial baseline ECSA (H-des) was about 26 m^2^ g^−1^, calculated from hydrogen desorption peak. In contrast, there are no obvious features of hydrogen adsorption/desorption for the cathode electrode of the melt blown membrane, assuming the 1, 2, 4-triazole additive migrated from the membrane to the surface of the catalyst where it was absorbed. This proves that the catalyst layer was contaminated, leading to ECSA losses and inferior fuel cell performance.

**Figure 4:**
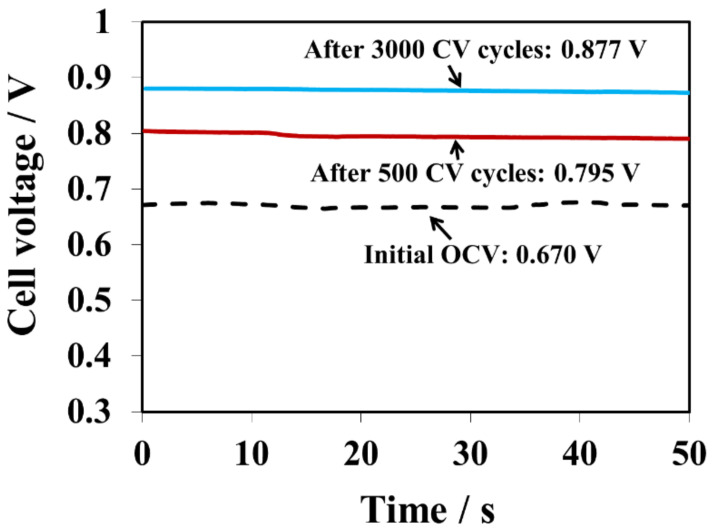
OCV changes before and after 500 and 3000 CV cycles for the melt blown membrane sample.

It is worthwhile to mention that the contaminated catalyst layers can be partially cleaned by CV cycling to remove the additives from the surface of catalyst. The clean-up efficacy depends on the amount of 1, 2, 4-triazole initially present in the membrane. As reported, some organic additive contaminants could be effectively removed by oxidation under higher potentials [17]. However, the CV cycling process may also cause the formation of Pt oxide, Pt particle sintering, Pt dissolution/re-deposition, and carbon support corrosion [18]. In this work, 3000 CV cycles under a wide potential window (0.1 to 1.2 V) were used as one of the approaches for triazole additive removal. Figure 4 and Table 1 show the OCV changes after 500, 1000, 1500, 2500, and 3000 CV cycles for the 1, 2, 4-triazole contaminated GDE. The OCV were compared with the baseline and the OCV ratio between the sample and the baseline was calculated. Clearly, the OCV gradually increased after every 500 CV cycles. For example, the OCV were 0.795V and 0.877V, which reached 82% and 90% of the baseline OCV value, after 500 and 3000 CV cycles, respectively. Therefore, CV cycling is a practical approach for removing the triazole additive from the catalyst layer. However, the OCV baseline value was not recoverable and no distinguishable features of hydrogen adsorption/desorption for the cathode electrode were observed, implying that the amount of 1, 2, 4-triazole additive in the membrane and GDEs was too high to be fully cleansed solely by CV cycling. It further demonstrates that a trace amount of triazole in the catalyst layer could have a huge impact on fuel cell performance.

**Figure 5:**
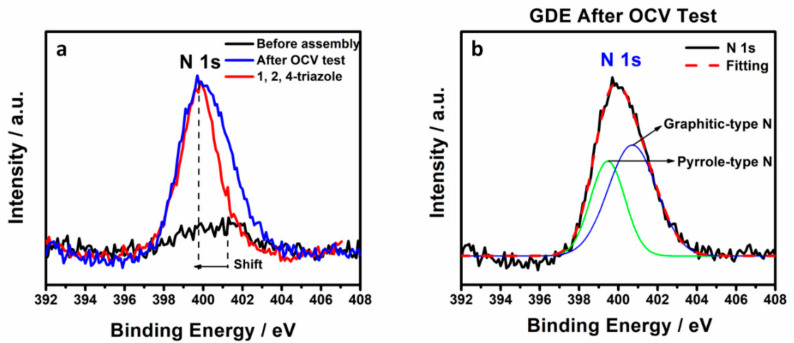
The core-level XPS spectra of N 1s for (**a**) untested, tested GDE and 1, 2, 4-triazole and (**b**) N1s XPS spectra for the tested GDE.

**Figure 6:**
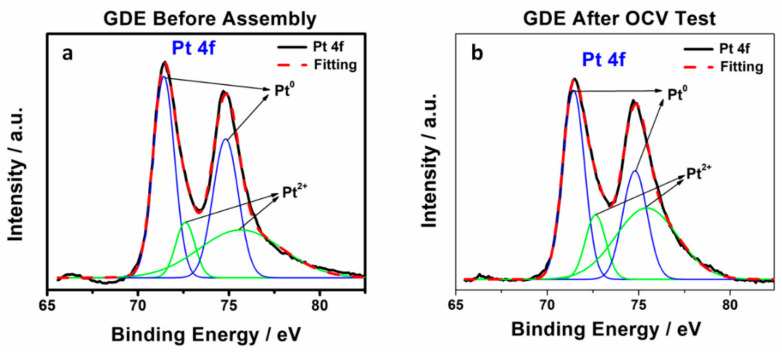
Core-level XPS spectra of Pt 4f of the cathodic GDE (**a**) before assembly and (**b**) after the OCV test.

The migration of triazole additive from membrane into the cathode catalyst layer was also confirmed by XPS. The survey spectra of untested and tested cathodic GDE assembly with melt blown membrane sample were collected in Figures S1 and S2, in which Pt 4f, C1s, O1s, N1s, F1s, and S2p orbitals can be observed and each individual element content was presented. As N is major element of the 1, 2, 4-triazole, it was considered as a GDE contamination indicator. The atomic ratio of N 1s: Pt 4f of GDE before and after fuel cell testing increased from 0.16 to 0.87 (see Table 2), indicating a non-negligible amount of additive moved out of the membrane into the GDE and remained in the cathode catalyst layer. The XPS trend is consistent with the CV results shown in Figure 3. The core-level XPS spectra of N 1s for untested, tested cathodic GDE and 1, 2, 4-triazole as a comparison are shown in Figure 5a. The tested cathodic GDE shows a negative shift of the N 1s binding energy and a relatively high peak intensity of N 1s, which has a similar N 1s binding energy as 1, 2, 4-triazole, compared to untested cathodic GDE, suggesting a considerable amount of N associated with triazole observed in the tested cathodic GDE. The N1s XPS spectra for tested cathodic GDE could be reasonably deconvoluted into two kinds of nitrogen functional groups: pyrrole-type N (400.2 eV) and graphitic-type N (401.1 eV) [19,20]. The deconvoluted N1s XPS spectra and calculated relative area of pyrrole-type N and graphitic-type N were shown in Figure 5b and Table 3, respectively, which identified the nature of triazole. Meanwhile, the core-level XPS spectra of Pt 4f is shown in Figure 6 and the principle peaks were attributed to Pt^0^ at 71.4 eV (4f_7/2_) and 74.7 eV (4f_5/2_), while 72.8 eV and 76.1 eV were assigned to Pt in 2+ state [21]. The results of different Pt species were calculated based on the deconvolution of these two kinds of Pt states and listed in Table 3. The relative area of Pt^0^ and Pt^2+^ was calculated to be 61% and 39%, respectively, for standard untested cathodic GDE. After fuel cell testing, the proportion of Pt^0^ on the surface decreased to 50%, while Pt^2+^ increased to 50%, indicating that the additional nitrogen containing coordination sites from 1, 2, 4-triazole possibly bonded to Pt. The XPS results proved that triazole additives migrated from the membrane into the catalyst and then attached to the Pt surface during OCV testing. 

### 3.2. Conditioning Behaviour of 1, 2, 4-Triazole Additive Contaminated GDE

**Figure 7:**
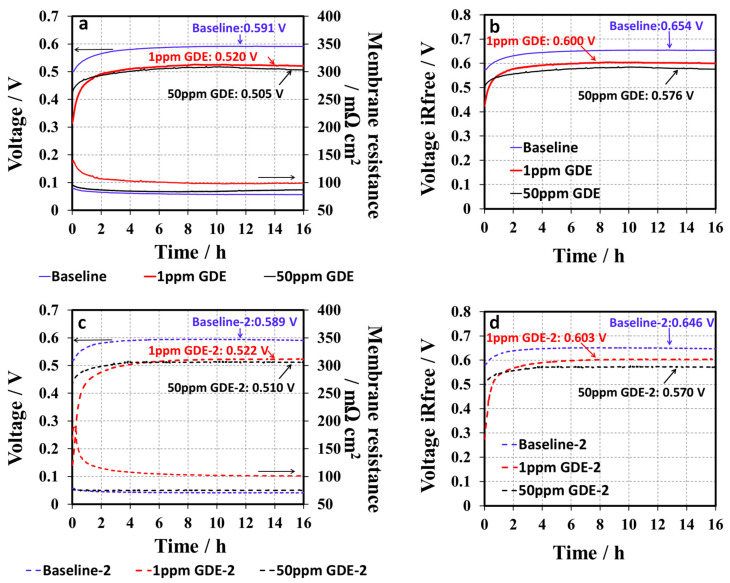
H_2_/air conditioning curves of (**a**) baseline, 1 ppm-GDE, and 50 ppm-GDE and (**c**) baseline-2, 1 ppm-GDE-2, and 50 ppm-GDE-2, obtained at 68 °C and 100% RH at a current density of 0.8 A cm^−2^ and their IR-compensated conditioning curves of (**b**) baseline, 1 ppm-GDE, and 50 ppm-GDE and (**d**) baseline-2, 1 ppm-GDE-2, and 50 ppm-GDE-2.

In order to further investigate the effects of 1, 2, 4-triazole additive on fuel cell performance, the triazole was intentionally introduced into GDEs by immerging the GDEs in either 1 ppm or 50 ppm 1, 2, 4-triazole additive solution for 48 h, which were named 1 ppm-GDE (GDE contaminated in 1 ppm of 1, 2, 4-triazole solution) and 50 ppm-GDE (GDE contaminated in 50 ppm of 1, 2, 4-triazole solution), respectively. The baseline was a standard GDE without any 1, 2, 4-triazole pre-treatment. Figure 7a shows the H_2_/air conditioning curves for baseline, 1 ppm, and 50 ppm GDE samples obtained at 68 °C and 100% RH at a current density of 0.8 A cm^−2^. Each GDE sample was tested with two duplicated samples for examining the experimental reproducibility, which was labelled as baseline-2, 1 ppm-GDE-2, and 50 ppm-GDE-2, respectively. The results are shown in Figure 7c. The repeatability of the test was acceptable because the difference in cell voltage between the two measurements (Figure 7a,c) was within 10 mV after conditioning. The cell voltage in the conditioning curves of the baseline GDEs gradually increased from 505 mV to 587 mV in the first 6 h and then reached a plateau, suggesting that the MEA be “well-conditioned.” Moreover, the high frequency resistance (HFR) collected by the current interrupt technique is mostly contributed from the membrane’s ionic resistance (see Figure 7a,c), which slightly decreased by ~15 mΩ cm^2^ during the conditioning, which is mainly attributed to the membrane hydration under 100% RH. Like the baseline, the conditioning curves of the 1 ppm-GDE showed a similar trend: the cell voltage gradually increased, and then reached its steady state (see Figure 7a). However, the 1 ppm-GDE sample showed inferior cell performance. The cell voltage for the 1 ppm-GDE sample was 190 mV and 70 mV lower than that of baseline at initial and after conditioning, respectively. For the 50 ppm-GDE, the performance drop was more obvious than the 1 ppm-GDE, since the cell performance was too low to draw a load at 20A. Alternatively, the 50 ppm-GDE sample was conditioned at a relatively low current of 0.4 A cm^−2^ for 16 h. After that, the conditioning curve was collected at an increased load, 0.8 A cm^−2^, as shown in Figure 7a. After conditioning, the cell voltages of the MEA samples at a current of 0.8 A cm^−2^ were in the order of 590 mV for the baseline, >520 mV for the 1 ppm-GDE, and >505 mV for the 50 ppm-GDE. The 50 ppm-GDE sample exhibited the lowest cell performance compared to the baseline and the 1 ppm-GDE. The absorption of 1, 2, 4-triazole in the catalyst layers led to a decrease in cell voltage, thus explaining the initial low fuel cell performance, while the removal of the contaminant from the cell by water generated from the electrochemical reaction or humidified gas streams resulted in a gradual improvement in cell performance. Additionally, the final cell performance for the contaminated GDE was still inferior to the baseline, implying that a certain amount of additive remained in the catalyst layer and the 50 ppm-GDE retained the most additive. IR-compensated conditioning curves were obtained by membrane resistance correction [22], as shown in Figure 7b,d. Similar to the non-corrected conditioning curves, IR-compensated conditioning curves showed the same trend: the cell voltages gradually went up, and then reached a plateau without further increases. It also demonstrated that HFR cannot be the main factor resulting in the differences of conditioning behavior between the MEAs. To further confirm the inferior cell performance of triazole doped GDEs, other in-situ characterizations were conducted and discussed in the following sections.

### 3.3. 2, 4-Triazole Additive Influence on Catalyst Layer

**Figure 8:**
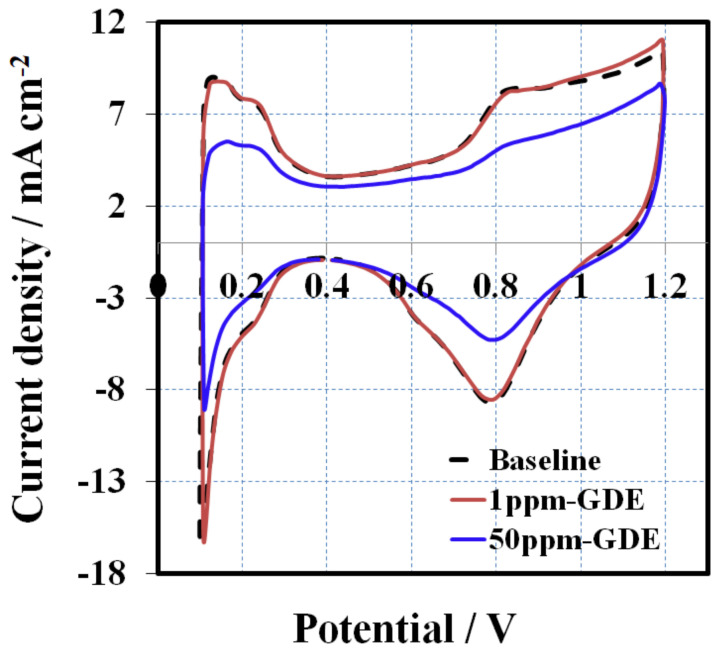
CVs for baseline, 1 ppm-GDE, and 50 ppm-GDE after conditioning at a current density of 0.8 A cm^−2^ at a scan rate of 50 mV s^−1^.

To conclude on whether the additive stuck to and contaminated the cathodic catalyst layer, all the MEAs underwent cyclic voltammetry (CV) to check the ECSA of the cathodic catalyst. The CV of the MEA with the membrane without additive (Nafion 211) served as the baseline. Figure 8 compares the CVs of the 1 ppm-GDE and the 50 ppm-GDE with the baseline after conditioning at a current density of 0.8 A cm^−2^ with a scan rate of 50 mV s^−1^. There were no obvious changes in ECSA between the 1 ppm-GDE and the baseline. However, a considerable difference in ECSA were observed, in which the peaks corresponding to adsorption/desorption of hydrogen for the 50 ppm-GDE (0.1–0.45 V) are smaller than that of the baseline, indicating a loss of Pt catalyst activity. After conditioning, ECSA (H-des) for the 50 ppm-GDE is 15 m^2^ g^−1^ whereas the baseline’s ECSA (H-des) is 26 m^2^ g^−1^, representing a ~40% ECSA loss compared to the baseline. The decline in ECSA can be ascribed to the leftover of additives in the catalyst layer and explains why the performance of the 50 ppm-GDE is lower than the baseline after conditioning.

### 3.4. 2, 4-Triazole Additive Influence on ORR Kinetics

**Figure 9:**
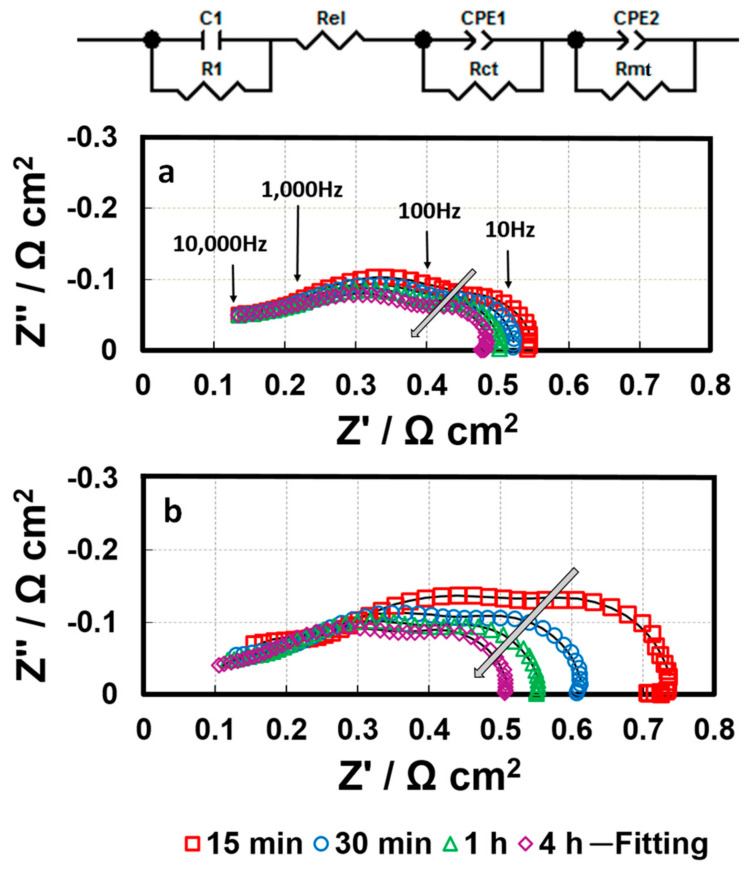
In-situ electrochemical impedance spectra of (**a**) baseline and (**b**) 1 ppm-GDE during conditioning at a current density of 0.8 A cm^−2^.

**Figure 10:**
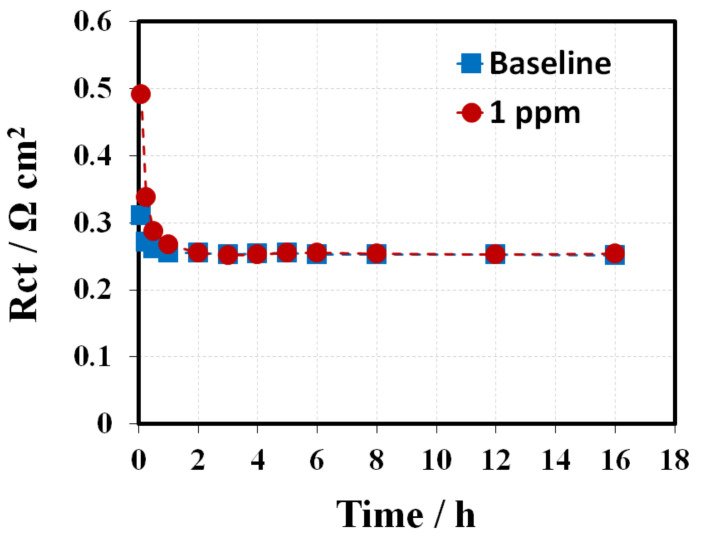
Calculated charge transfer resistance of the baseline and the 1 ppm-GDE during conditioning at a current density of 0.8 A cm^−2^.

**Figure 11:**
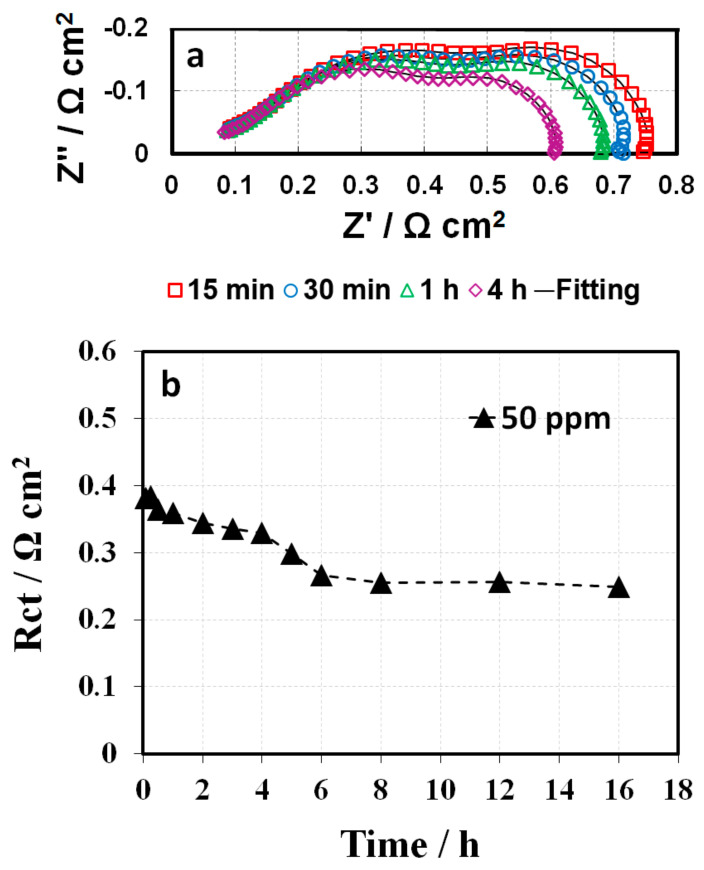
(**a**) In-situ electrochemical impedance spectra and (**b**) calculated charge transfer resistance of the 50 ppm-GDE during conditioning at a current density of 0.8 A cm^−2^.

During conditioning, in-situ EIS was conducted to improve understanding of the mechanism behind the impacts of triazole additives on fuel cell performance. Generally, when the cell is operated at high current densities, a double semicircle in the Nyquist plot could be observed, which is attributed to the sum of the charge transfer resistance (Rct) and mass transport resistance (Rmt). Figure 9 shows the spectrum (Nyquist plots) of the baseline and the 1 ppm-GDE, including one high frequency (HF) capacitive loop and one medium frequency (MF) capacitive loop, as well as one low frequency (LF) capacitive loop and one LF inductive loop [23]. The small HF capacitive loop can be fitted by a contact resistance R1 in parallel with a contact capacitance C1, associated with either the electronic contact impedance or the ionic ohmic drop inside the active layer [24]. The MF impedance arc mainly corresponds to the charge transfer resistance associated with the ORR, and the LF domain is predominantly attributed to the mass transfer resistance, representing the resistance to mass transfer in the gas phase within the gas diffusion layer and the catalyst layer. Based on the essential feature of fuel cell reactions and the observed electrochemical phenomena, the MF and LF loops can be fitted using the equivalent circuit shown in Figure 9, consisting of a charge transfer resistance Rct in parallel with a constant phase element (CPE1) and a mass transfer resistance Rmt in parallel with a constant phase element (CPE2), respectively [25]. With increasing conditioning time, the MF capacitive loop changes significantly. In contrast, there was negligible variation in the LF inductive loops. Therefore, the fitting for LF inductive loops was not conducted in this work. Figure 9 and Figure 10 present the fitting curves (solid line) and fitting results, respectively. The Rct for the baseline have a ~15% decrease compared to their initial values during the entire conditioning period. In comparison, the initial Rct for the 1 ppm-GDE was 180 mΩ cm^2^ (~50%) higher than that of the baseline. The results implied the triazole additive bonding/poisoning with/of the Pt catalyst, which led to a significant increment in Rct at the beginning. Meanwhile, the Rct for 1 ppm-GDE exhibited a quick decrease from ~490 mΩ cm^2^ to ~250 mΩ cm^2^ in the first 4 h, accounting for more than 50%. The results indicate that the triazole additive bonded on Pt catalyst surface could be washed away either by humidified gas streams or the water generated from the electrochemical reaction [25,26]. The Rct gradually reduced and then nearly approached the baseline after a certain amount of additive was rinsed off from the catalyst surface by water. Alternatively, the 50 ppm-GDE sample needed to be conditioned in two consecutive steps, at a current density of 0.4 A cm^−2^ for 16 h and then at a current density of 0.8 A cm^−2^ for another 16 h, due to the triazole additive severe poisoning of the catalyst layer. As a result, Rct decreased gradually and reached the same value as the baseline (Figure 11), which suggests that contaminant removal requires additional energy or extended conditioning time. Moreover, the inferior voltage compared to the baseline after conditioning suggests that the anode might be contaminated, or the additive remains in GDLs. Further study is ongoing to determine the hypothesis.

## 4. Conclusions

In this study, the 1, 2, 4-triazole contaminated membrane was tested under OCV conditions and 1, 2, 4-triazole contaminated cathode GDE were investigated under fuel cell conditioning conditions. The samples were characterized and diagnosed in PEMFC using a variety of techniques, such as ECSA, in-situ EIS, UV-Vis spectra, and XPS. The overall results presented in this report demonstrated that 1, 2, 4-triazole as an additive does migrate from the membrane and contaminates the catalyst layer. The release of extra additive caused lower OCV, longer conditioning time, and inferior fuel cell performance due to catalyst layer contamination. Conditioning and CV cycling are two practical approaches to remove the migrated additive partially/completely from catalyst layer, which depends on the amount of additive remaining in the membrane. However, in the worst-case scenario, the migrated additive could not be removed completely, leading to severe inferior fuel cell performance. From a manufacturing perspective, this work confirms that a post-treatment step following melt-blowing processing of the membrane to remove organic additives from the membrane is highly recommended. The findings in this work at the same time provides some insights on quality control of membrane for manufacturers, to control and minimize the amount of additive remaining in the membranes in order to achieve the best fuel cell performance.

## Figures and Tables

**Table 1:** OCV changes and increments after CV cycles.

CV Cycle Numbers	OCV/V	OCV Ratio (Sample: Baseline)
0	0.670	0.69
500	0.795	0.82
1000	0.805	0.83
1500	0.814	0.84
2500	0.876	0.90
3000	0.877	0.90
Baseline OCV	0.972	-

**Table 2:** Calculated atomic ratio of N 1s: Pt 4f.

Cathodic GDE	Pt 4f (Atomic Percentage %)	N 1s (Atomic Percentage %)	Ratio (N 1s:Pt 4f)
Before Assembly	4.73	0.78	0.16
After Test	3.74	3.26	0.87

**Table 3:** Calculated relative area of N and Pt.

Cathodic GDE	Pt Species	Relative Area (%)
Before Assembly	Pt^0^	61
	Pt^2+^	39
	graphitic-type N	100
After Test	Pt^0^	50
	Pt^2+^	50
	pyrrole-type N	38
	graphitic-type N	62

## Supplementary Materials

The following are available online at https://www.mdpi.com/2077-0375/10/11/301/s1, Figure S1: XPS survey spectra of untested standard GDE. Figure S2: XPS survey spectra of the tested GDE assembly with melt blown membrane sample.

## References

[1] Peighambardoust S., Rowshanzamir S., Amjadi M. (2010). Review of the proton exchange membranes for fuel cell applications. Int. J. Hydrog. Energy.

[2] Kraytsberg A., Ein-Eli Y. (2014). Review of Advanced Materials for Proton Exchange Membrane Fuel Cells. Energy Fuels.

[3] Thompson S.T., James B.D., Huya-Kouadio J.M., Houchins C., DeSantis D.A., Ahluwalia R., Wilson A.R., Kleen G., Papageorgopoulos D. (2018). Direct hydrogen fuel cell electric vehicle cost analysis: System and high-volume manufacturing description, validation, and outlook. J. Power Sources.

[4] Lai Y.-H., Mittelsteadt C.K., Gittleman C.S., Dillard D.A. (2009). Viscoelastic Stress Analysis of Constrained Proton Exchange Membranes Under Humidity Cycling. J. Fuel Cell Sci. Technol..

[5] Mokrini A. Study of azoles as bifunctional ddditives for proton exchange membranes melt-processing from LSC and SSC perfluorosulfonic acid ionomers. Proceedings of the 2014 ECS and SMEQ Joint International Meeting.

[6] Kim J.-D., Jun M.-S. (2012). Nafion-1, 2, 3-Triazole Blend Membranes for High Temperature PEMFCs. Fuel Cells.

[7] Subbaraman R., Ghassemi H., Zawodzinski T.A. (2007). 4, 5-Dicyano-1H-[1, 2, 3]-Triazole as a Proton Transport Facilitator for Polymer Electrolyte Membrane Fuel Cells. J. Am. Chem. Soc..

[8] Kim J.-D., Mori T., Hayashi S., Honma I. (2007). Anhydrous Proton-Conducting Properties of Nafion–1, 2, 4-Triazole and Nafion–Benzimidazole Membranes for Polymer Electrolyte Fuel Cells. J. Electrochem. Soc..

[9] Subbaraman R., Ghassemi H., Zawodzinski T. (2009). Triazole and triazole derivatives as proton transport facilitators in polymer electrolyte membrane fuel cells. Solid State Ionics.

[10] Song M.-K., Zhu X., Liu M. (2013). A triazole-based polymer electrolyte membrane for fuel cells operated in a wide temperature range (25–150 °C) with little humidification. J. Power Sources.

[11] Kreuer K., Fuchs A., Ise M., Spaeth M., Maier J. (1998). Imidazole and pyrazole-based proton conducting polymers and liquids. Electrochim. Acta.

[12] Schuster M.F.H., Meyer W.H. (2003). Anhydrous Proton-Conducting Polymers. Annu. Rev. Mater. Res..

[13] Mokroni A. (2017). Process for Producing ion Exchange Membranes by Melt-Processing of Acidic PFSA Ionomers. U.S. Patent.

[14] Wang L., Zhu M.-J., Yang F.-C., Gao C.-W. (2012). Study of a Triazole Derivative as Corrosion Inhibitor for Mild Steel in Phosphoric Acid Solution. Int. J. Corros..

[15] http://www.scribner.com/faq/9-what-can-cause-a-low-ocv-open-circuit-voltage/.

[16] Zhang Z.-H., Tizzard G.J., Williams J.A.G., Goldup S.M. (2020). Rotaxane PtII-complexes: Mechanical bonding for chemically robust luminophores and stimuli responsive behaviour. Chem. Sci..

[17] Gasteiger H.A., Kocha S.S., Sompalli B., Wagner F.T. (2005). Activity benchmarks and requirements for Pt, Pt-alloy, and non-Pt oxygen reduction catalysts for PEMFCs. Appl. Catal. B Environ..

[18] Zhang Y., Chen S., Wang Y., Ding W., Wu R., Li L., Qi X., Wei Z. (2015). Study of the degradation mechanisms of carbon-supported platinum fuel cells catalyst via different accelerated stress test. J. Power Sources.

[19] Cheng J., Li Y., Mei A., Huang X., Wang Q., Shen P.K. (2015). Highly stable electrocatalysts supported on nitrogen-self-doped three-dimensional graphene-like networks with hierarchical porous structures. J. Mater. Chem. A.

[20] He C., Shen P.K. (2013). Synthesis of the nitrogen-doped carbon nanotube (NCNT) bouquets and their electrochemical properties. Electrochem. Commun..

[21] Bera P., Priolkar K.R., Gayen A., Sarode P.R., Hegde M.S., Emura S., Kumashiro R., Jayaram V., Subbanna G.N. (2003). Ionic Dispersion of Pt over CeO2by the Combustion Method: Structural Investigation by XRD, TEM, XPS, and EXAFS. Chem. Mater..

[22] Xie Z., Zhao X., Adachi M., Shi Z., Mashio T., Ohma A., Shinohara K., Holdcroft S., Navessin T. (2008). Fuel cell cathode catalyst layers from “green” catalyst inks. Energy Environ. Sci..

[23] Yuan X.-Z., Song C., Wang H., Zhang J. (2010). EIS Diagnosis for PEM Fuel Cell Performance. Electrochemical Impedance Spectroscopy in PEM Fuel Cells.

[24] Antoine O., Bultel Y., Durand R. (2001). Oxygen reduction reaction kinetics and mechanism on platinum nanoparticles inside Nafion^®^. J. Electroanal. Chem..

[25] Zhao N., Xie Z., Shi Z. (2018). Understanding of Nafion Membrane Additive Behaviors in Proton Exchange Membrane Fuel Cell Conditioning. J. Electrochem. Energy Convers. Storage.

[26] Zhao N., Yuan X., Girard F., Wang K., Li J., Shi Z., Xie Z. (2019). Effects of Membrane Additives on PEMFC Conditioning. ChemistrySelect.

