# Supplementary material for: Effects of 1, 2, 4-Triazole Additive on PEM Fuel Cell Conditioning
Source: Membranes (Basel). 2020 Oct 22;10(11):301. doi: 10.3390/membranes10110301 (PMC7690283; doi:10.3390/membranes10110301)
Supplement: Supplementary file 1 [file membranes-10-00301-s001.pdf]

Supporting Information

Effects of 1, 2, 4-Triazole Additive on PEM Fuel Cell Conditioning

Nana Zhao [1*], Zhiqing Shi [1*], Régis Chenitz [2], Francois Girard [1], Asmae Mokrini [2*]

[1] Energy, Mining & Environment Research Centre, National Research Council Canada, 4250 Wesbrook Mall, Vancouver, B.C. Canada, V6T 1W5

[2]Automotive and Surface Transportation Research Centre, National Research Council Canada, 75 de Mortagne, Boucherville, Québec, Canada, J4B 6Y4

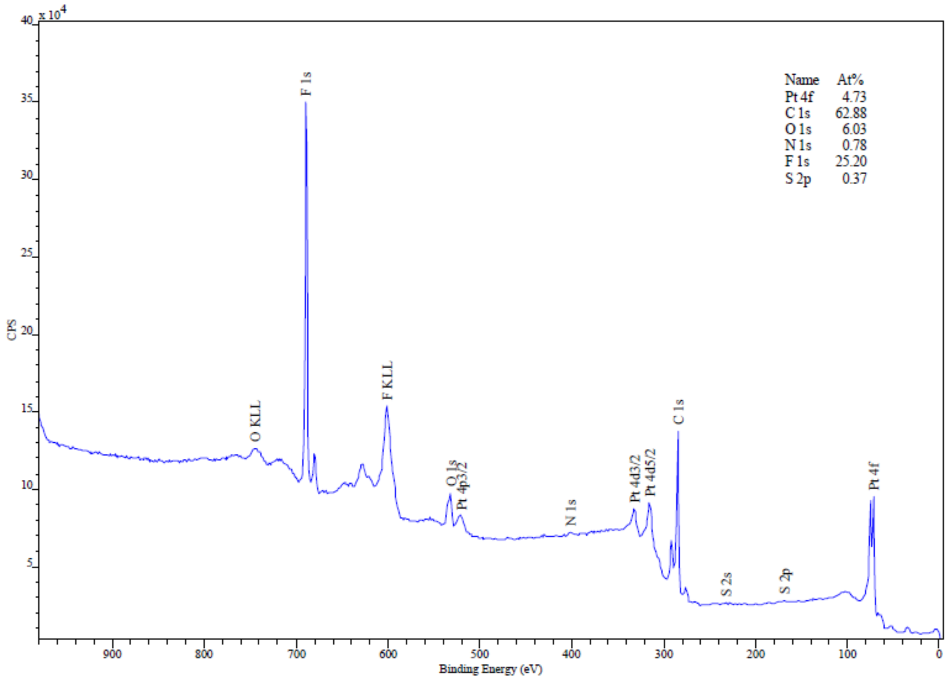

Figure S1 XPS survey spectra of untested standard GDE.

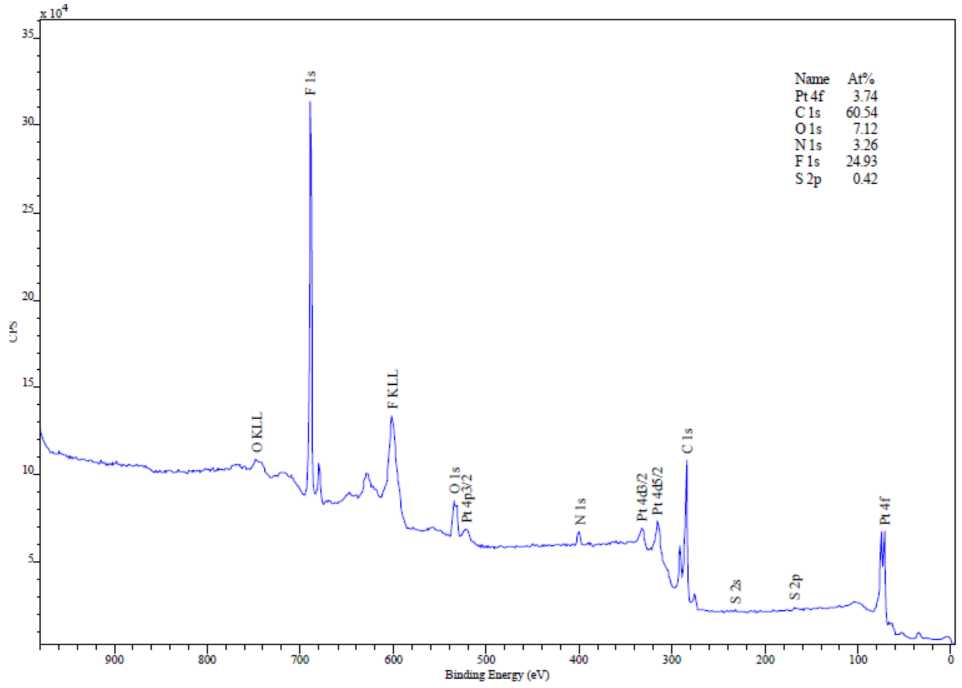

Figure S2 XPS survey spectra of the tested GDE assembly with melt blown membrane sample.
